# Supplementary material for: Taxon Cycles in Neotropical Mangroves
Source: Plants (Basel). 2023 Jan 5;12(2):244. doi: 10.3390/plants12020244 (PMC9864432; doi:10.3390/plants12020244)
Supplement: Supplementary file 1 [file plants-12-00244-s001.zip › plants-2092593-supplementary.pdf]

Taxon cycles in Neotropical mangroves

Valentí Rull

Botanic Institute of Barcelona (CSIC)
Consejo Superior de Investigaciones Científicas (CSIC)
08038 Barcelona, Spain
vrull@csic.es

Supplementary Material

Table S1. Eocene to Pliocene *Pelliciera* (represented by *Lanagiopollis crassa* and *Psilatricolporites crassus*) and *Rhizophora* (represented by *Zonocostites ramonae* and other species of this genus) records from the Neotropics, with indication of the relative abundance according to the original data reported in the corresponding papers (+ present, - absent or not mentioned). Only *Rhizophora* records within the distribution area of *Pelliciera* are considered. Numbers (N) correspond to localities depicted in Figures S1 to S4. Age: Eo, Eocene; Oli, Oligocene; Mio, Miocene; Plio, Pliocene; E, Early; M, Middle; L Late. Countries: BRA, Brasil; COL, Colombia; CRI, Costa Rica; CUB, Cuba; GUA, Guatemala; GUY, Guyana; HAI, Haiti; JAM, Jamaica; MEX, Mexico; PAN, Panamá; PER, Perú; PUR, Puerto Rico; SUR, Surinam; TRI, Trinidad; VEN, Venezuela.

N	Site/area	Country	Age	*Pelliciera*	*Rhizophora*	References
1	Columbus	TRI	Plio	+	+	Lamy (1986)
2	Orinoco	VEN	Plio	+	+	Pocknall et al. (2001)
3	Talamanca	CRI	Plio	+	+(?)	Graham & Dilcher (1998)
4	Montañita	VEN	Plio	+	+	Lorente (1986)
5	Jocotán	GUA	Plio	-	≤100%	Graham (1998)
6	Barinas	VEN	Mio-Plio	+	+	Bermúdez et al. (2017)
7	Gatun	PAN	Mio-Plio	-	≤80%	Graham (1990b, 1991)
8	Urumaco	VEN	Mio-Plio	+	≤70%	Lorente (1986); Hambalek et al. (1994)
9	Tig-141X	VEN	L Mio	+	≤20%	Lorente (1986)
10	Veracruz	MEX	L Mio	-	≤96%	Graham (1975, 1976)
11	Zamuro-1X	VEN	L Mio	+	≤50%	Lorente (1986)
12	Guanipa-1X	VEN	L Mio	+	≤70%	Lorente (1986)
13	Hervidero-1X	VEN	L Mio	+	≤60%	Lorente (1986)
14	Cucaracha	PAN	M Mio	-	≤60%	Graham (1988b)
15	Yopal	COL	M Mio	-	+	Dueñas & Van der Hammen (2007)
16	TJ	VEN	E Mio	+	+	Rull (2001)
17	Sheroli	CRI	E Mio	-	≤5%	Graham (1987)
18	Gold Hill	PAN	E Mio	-	Common	Graham (1988a)
19	SZZ-119X	VEN	E Mio	-	≤60%	Lorente (1986)
20	SCZ-124X	VEN	E Mio	+	≤60%	Lorente (1986)
21	IZZ-100X	VEN	E Mio	-	≤20%	Lorente (1986)
22	Cascadas	PAN	E Mio	≤3%	70-90%	Graham (1989)
23	Saltarín	COL	E/M Mio	+	Abundant	Jaramillo et al. (2017)
24	NZZ-154/160	VEN	E/M Mio	+	≤80%	Lorente (1986)
25	Los Pobres-1	VEN	E/M Mio	+	≤40%	Lorente (1986)
26	Pariaguán-1X	VEN	E/M Mio	≤10%	≤90%	Lorente (1986)
27	Panchita-1X	VEN	E/M Mio	+	≤90%	Lorente (1986)
28	SDZ-117X	VEN	E/M Mio	-	≤30%	Lorente (1986)
29	LSJ-3310	VEN	E/M Mio	+	≤60%	Lorente (1986)
30	LS-992B	VEN	E/M Mio	+	≤40%	Lorente (1986)
31	LL-370	VEN	E/M Mio	+	≤30%	Lorente (1986)
32	Pozón-SX	VEN	E/M Mio	≤10%	-	Lorente (1986)
33	CO-85	TRI	Mio	+	Abundant	Germeraad et al. (1968)
34	B-188	VEN	E Mio	≤3%	≤80%	Lorente (1986)
35	Alliance	SUR	Mio	+	+	Wijmstra (1969)
36	SLA	VEN	Mio	-	Abundant	Rull (1992)
37	Culebra-1X	VEN	Mio	+	≤80%	Lorente (1986)
38	Catatumbo-1X	VEN	Mio	≤10%	≤40%	Lorente (1986)
39	OG-1X	VEN	Mio	+	≤80%	Lorente (1986)
40	Mariñame	COL	E/M Mio	+	≤70%	Hoorn (1994)
41	Chorros	COL	Mio	+	≤20%	Hoorn et al. (2022)
42	La Frontera	PER	Mio	+	-	Parra et al. (2020)
43	105-AM	COL	E/M Mio	+	+	Jaramillo et al. (2017)
44	Pará	BRA	Mio	≤2%	≤40%	Antonioli et al. (2015); Aguilera et al. (2017)
45	Amazon	BRA	Oli-Mio	+	+	De Boer et al. (1965)
46	Maranhão	BRA	Oli-Mio	+	+	Regali et al. (1974)
47	Banneirinhas	BRA	Oli-Mio			Regali et al. (1985)
48	Coastal Plain	SUR	Oli-Mio	+	+	Amstelveen (1971)
49	Falcón	VEN	Oli-Mio	-	Abundant	Rull & Poumot (1997)
50	Maturín	VEN	Oli-Mio	+	-	Helenes & Cabrera (2002)

51	Chama	VEN	Oli-Mio	-	Common	Rull (1997a, 2002)
52	Shelter Belt	GUY	Oli-Mio	≤5%	≤90%	Van der Hammen & Wijmstra (1964); Wijmstra (1968)
53	Planeta Rica	COL	L Oli-E Mio	-	Abundant	Dueñas (1980)
54	Chafurray	COL	Oli-Mio	+	Abundant	Germeraad et al. (1968)
55	Furrial	VEN	Oli-Mio	+	+	Fasola et al. (1991)
56	Simojovel	MEX	Oli-Mio	≤2.5%	>95%	Langenheim et al. (1967); Graham (1999)
57	Carbón	VEN	Oli	≤25%	≤50%	Lorente (1986)
58	Friata-1X	VEN	Oli	+	+	Lorente (1986)
59	Lares	PRI	Oli	≤2%	≤95%	Graham & Jarzen (1969)
60	Pozón-SX	VEN	Oli	≤10%	-	Lorente (1986)
61	COT-1X	VEN	Oli	+	Common	Rull (2003)
62	Tig-141X	VEN	Oli	+	-	Lorente (1986)
63	Concentración	COL	L Eo/E Oli	≤47%	-	Ochoa et al. (2012)
64	Delicias	VEN	Eo/Oli	+	-	Colmenares & Teran (1993)
65	Bogotá	COL	L Eo	≤15%	+	Ochoa et al. (2012)
66	COT-1X	VEN	L Eo	Com	+	Rull (2003)
67	Alcalde Díaz	PAN	M/L Eo	≤11%	≤10%	Graham (1985)
68	Tarra	VEN	M/L Eo	Common	+	Rull (1997b)
69	Paz del Rio	COL	M Eo	Abundant	-	Germeraad et al. (1968)
70	VLC	VEN	M Eo	≤60%	-	Rull (1998, 2002)
71	Chapelton	JAM	M Eo	Common	-	Graham (1977)
72	Sagu	COL	M Eo	Common	-	De la Parra et al. (2021)
73	Icotea	VEN	M Eo	Abundant	-	Germeraad et al. (1968)
74	Piñalerita	COL	M Eo	Common	+	Jaramillo & Dilcher (2001)
75	Boscán	VEN	M Eo	+	+	Colmenares (1988)
76	Rubio/Lora	VEN	E/M Eo	+	-	Germeraad et al. (1968); Pocknall & Erlich (2020)
77	Maracaibo	VEN	Eo	Abundant	-	Rull & Poumot (1997)
78	Nuevo Mundo	COL	Eo	Abundant	-	Rodríguez-Forero et al. (2012)
79	Maché	VEN	E Eo	Common	-	Rull (1999, 2000, 2002)

Figures S1 to S4. Maps showing the location of the studied sites grouped by geological epochs. Raw data and numbers from Table S1.

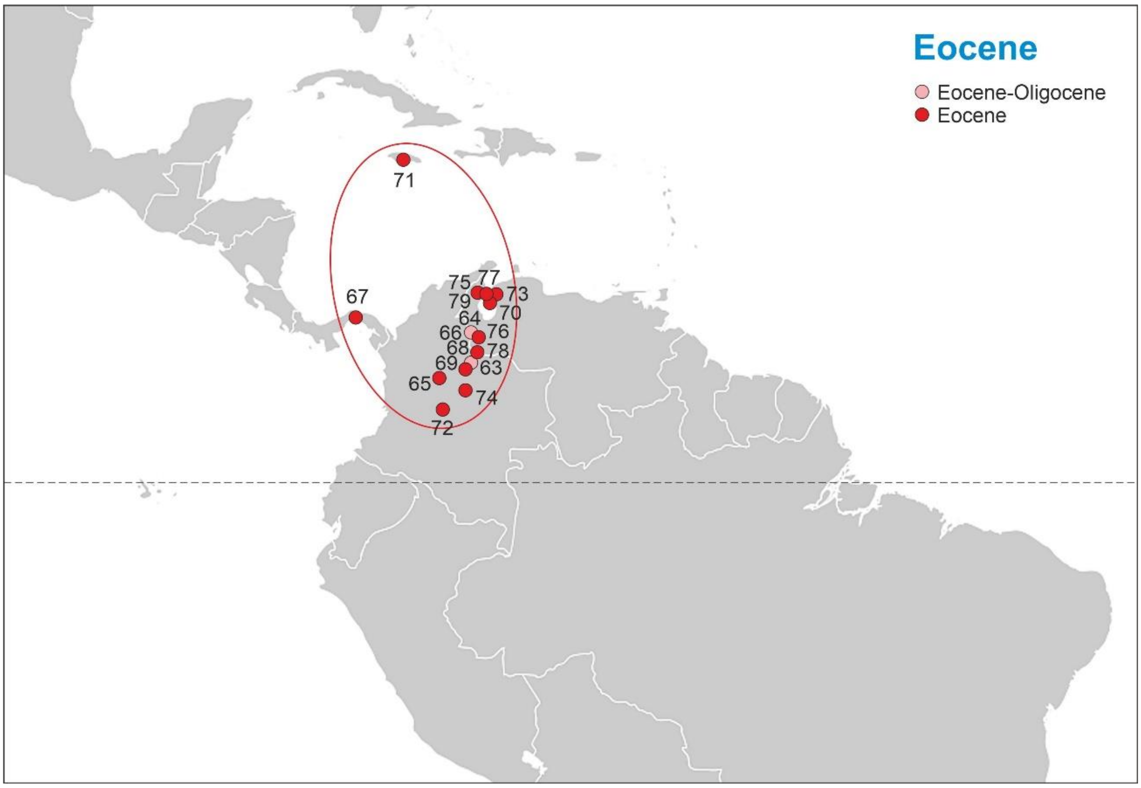

Figure S1. Eocene.

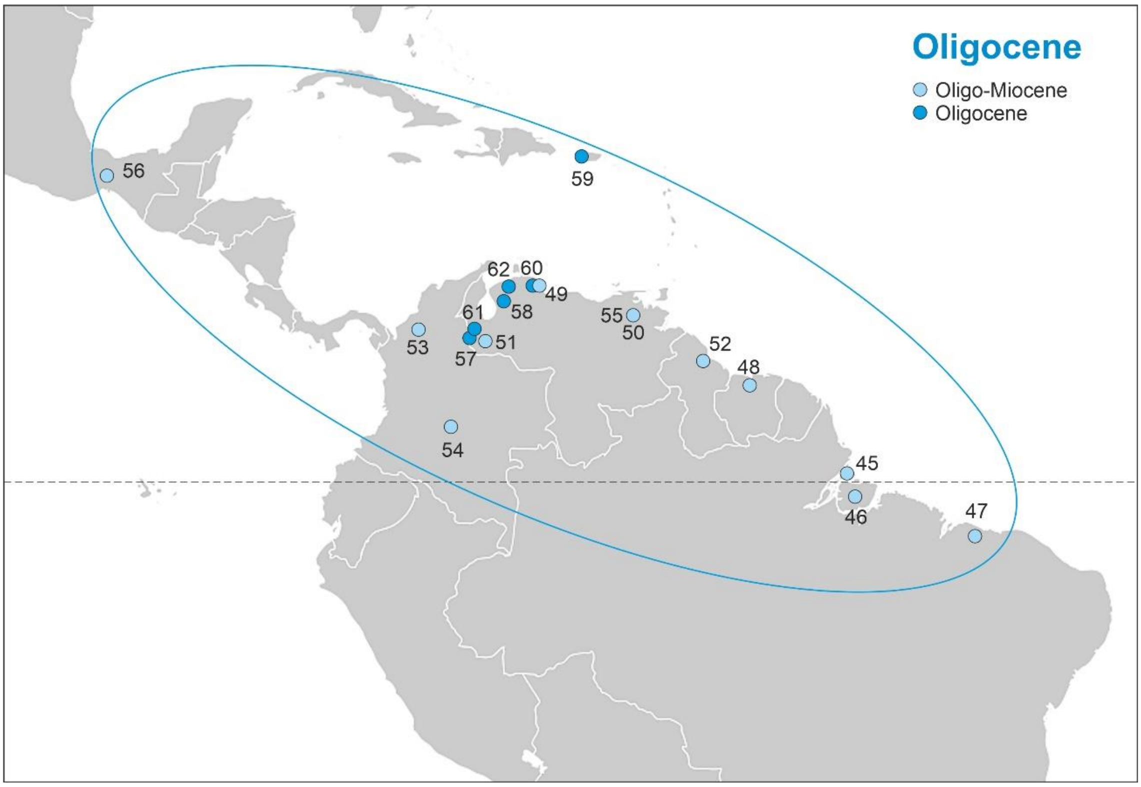

Figure S2. Oligocene.

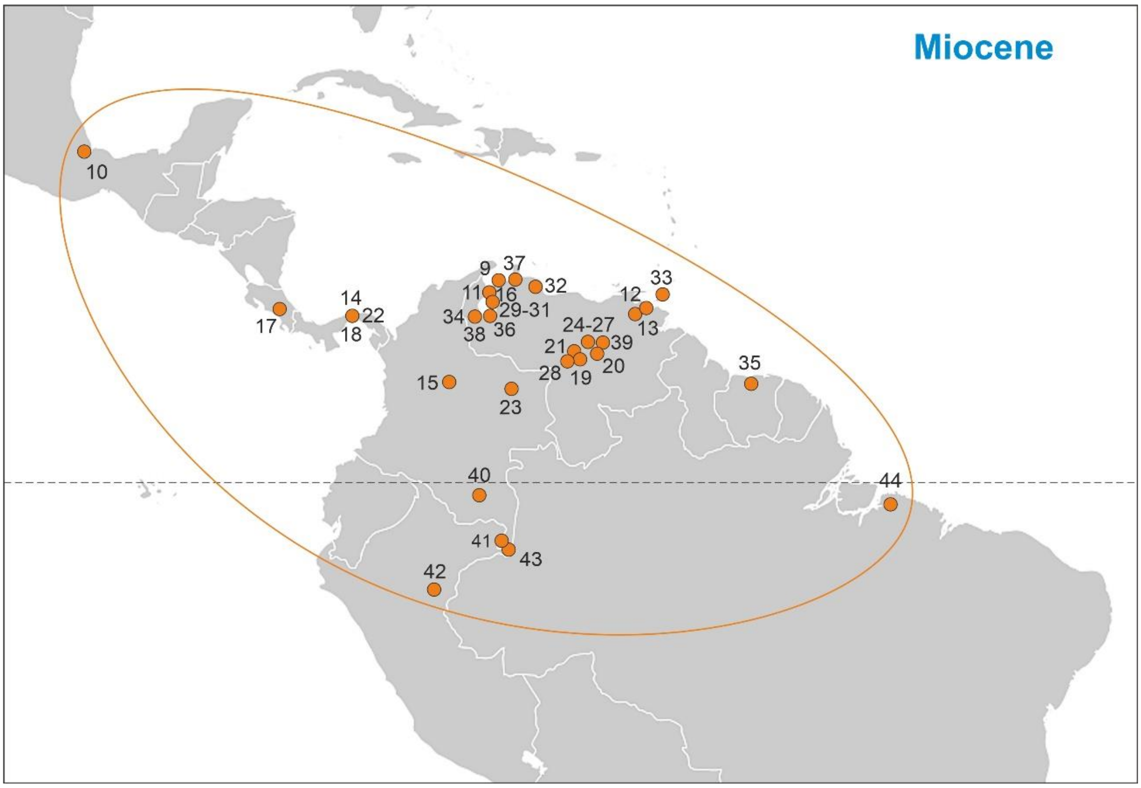

Figure S3. Miocene.

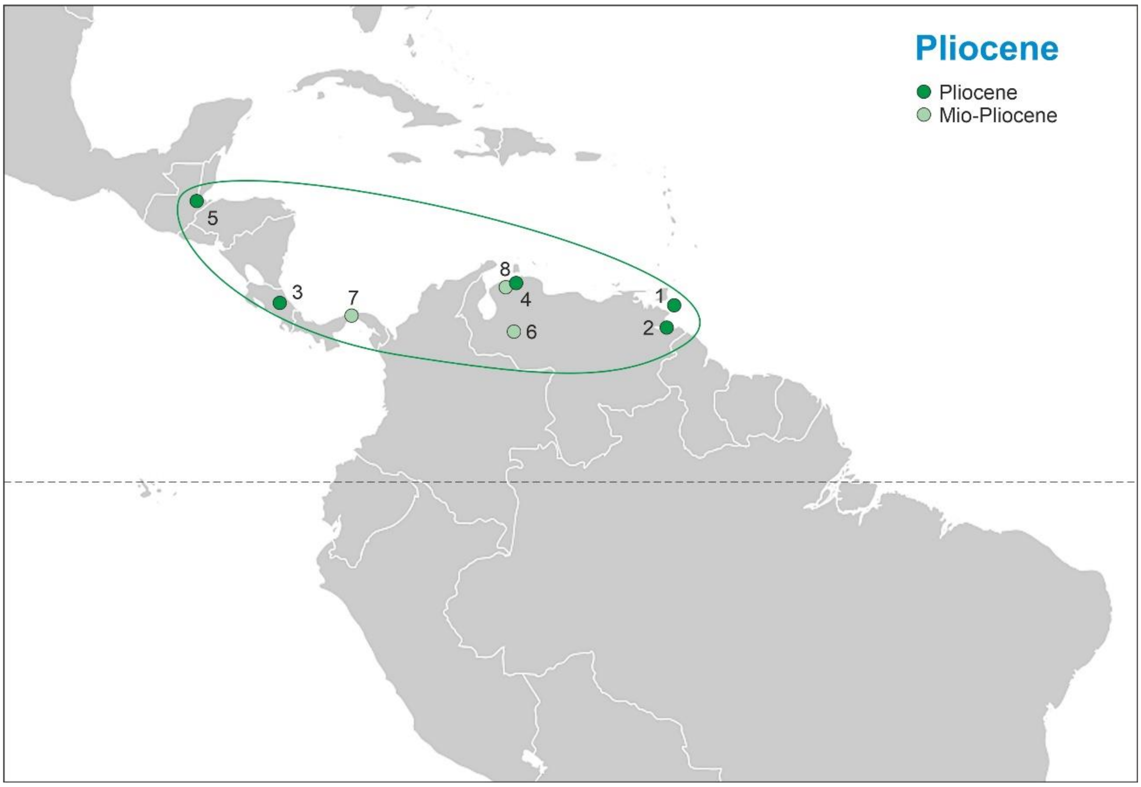

Figure S4. Pliocene.

References

Aguilera, O., Silva, G.O.A., Lopes, R.T., Machado, A.S., dos Santos, T.M., Marques, G., et al. 2017. Neogene Proto-Caribbean porcupinefishes (Diodontidae). PLos ONE 12, e0181670.

Amstelveen, A.L.E. 1971. A palynostratigraphical correlation of seven test wells in the Coastal Plain of Surinam. Geologie en Mijnbouw 21, 177-182.

Antonioli, L., Távora, V.A., Dino, R. 2015. Palynology of carcinolites and limestones from the Baunilha Grande Ecofacies of the Pirabas Formation (Miocene of Pará state, northeastern Brazil). Journal of South American Earth Sciences 62, 134-147.

Bermúdez, M.A., Hoorn, C., Bernet, M., Carrillo, E., van der Beek, P.A., Garver, J.I., et al. 2017. The detrital record of late-Miocene to Pliocene surface uplift and exhumation of the Venezuelan Andes in the Maracaibo and Barinas foreland basins. Basin research 29, 370-395.

Colmenares, O.A. 1988. A palynological study of samples from three wells of the Boscan Field, Venezuela. Revista Técnica INTEVEP 8, 83-97.

Colmenares, O.A., Teran, L. 1993. A biostratigraphic study of Paleogene sequences in southwestern Venezuela. Palynology 17, 67-89.

De Boer, N.P., Van der Hammen, T., Wijmstra, T.A. 1965. A palynological study on the age of some borehole samples from the Amazonas Delta area, northeastern Brazil. Geologie en Mijnbouw 44, 254-258.

De la Parra, F., Pinzón, D., Mantilla-Duran, F., Rodriguez, G., Caballero, V. 2021. Marine-lacustrine systems during the Eocene of northern South America – palynological evidence from Colombia. Journal of South American Earth Sciences 108, 103188.

Dueñas, H. 1980. Palynology of Oligocene-Miocene strata of borehole Q-E-22, Planeta Rica, northern Colombia. Review of Palaeobotany and Palynology 30, 313-328.

Dueñas, H., Van der Hammen, T. 2007. Significado geológico y asociaciones palinológicas de las formaciones Diablo Inferior (Mioceno Tardío) y San Fernando Superior (Mioceno Medio), piedemonte Cuenca de los Llanos Orientales, Colombia. Revista de la Academia Colombiana de Ciencias 31, 481-498.

Fasola, A., et al. 1991. Late Cretaceous palynological assemblages from El Furrial area wells. Revista Técnica INTEVEP 11, 3-13.

Germeraad, J.H.; Hopping, C.A.; Muller, J. 1968. Palynology of Tertiary sediments from tropical areas. Review of Palaeobotany and Palynology 6, 189-348.

Graham, A. 1975. Late Cenozoic evolution of tropical lowland vegetation in Veracruz, Mexico. Evolution 29, 723-735.

Graham, A. 1976. Studies in Neotropical botany. II. The Miocene communities of Veracruz, Mexico. Annals of the Missouri Botanical Garden 63, 787-842.

Graham, A. 1977. New records of *Pelliciera* (Theaceae/Pellicieriaceae) in the Tertiary of the Caribbean. Biotropica 9, 48–52.

Graham, A. 1985. Studies in Neotropical paleobotany. IV. The Eocene communities of Panama. Annals of the Missouri Botanical Garden 72, 504-534.

Graham, A. 1987. Miocene communities and paleoenviroments of southern Costa Rica. American Journal of Botany 74, 1501-1518.

Graham, A. 1988a. Studies in Neotropical paleobotany. V. The lower Miocene communities of Panama-the Culebra Formation. Annals of the Missouri Botanical Garden 75, 1440-1466.

Graham, A. 1988b. Studies in Neotropical paleobotany. VI. The lower Miocene communities of Panama-the Cucaracha Formation. Annals of the Missouri Botanical Garden 75, 1467-1479.

Graham, A. 1989. Studies in Neotropical paleobotany, VII. The lower Miocene communities of Panama-the La Boca Formation. Annals of the Missouri Botanical Garden 76, 50-66.

Graham, A. 1990b. New angiosperm records from the Caribbean Tertiary. American Journal of Botany 77, 897-910.

Graham, A. 1991. Studies in Neotropical botany. X. The Pliocene communities of Panama-composition, numerical representations, and paleocommunity paleoenvironmental reconstructions. Annals of the Missouri Botanical Garden 78, 465-475.

Graham, A. 1998. Studies in Neotropical botany. XI. Late Tertiary vegetation and environments of southeastern Guatemala: palynofloras from the Mio-Pliocene Padre Miguel Group and the Pliocene Herrería Formation. American Journal of Botany 85, 1409-1425.

Graham, A. 1999. Studies in Neotropical paleobotany. XIII. An Oligo-Miocene palynolflora from Simojovel (Chiapas, Mexico). American Journal of Botany 86, 17-31.

Graham, A., Dilcher, D.L. 1998. Studies in Neotropical botany. XII. A palynoflora from the Pliocene Rio Banano Formation of Costa Rica and the Neogene vegetation of Mesoamerica. American Journal of Botany 85, 1426-1438.

Graham, A., Jarzen, D.M. 1969. Studies on Neotropical paleobotany. I. The Oligocene communities of Puerto Rico. Annals of the Missouri Botanical Garden 56, 308-357.

Hambalek, N., Rull, V., DiGiacomo, E., Gamero, M.L. 1994. Evolución paleoecológica y paleoambiental de la secuencia del Neógeno en el Surco de Urumaco, Estado Falcón. Estudio palinológico y litológico. Boletín de la Sociedad Venezolana de Geólogos 1-2, 7-19.

Helenes, J., Cabrera, D. 2002. Oligo-Miocene palynomorph assemblages from eastern Venezuela. Palynology 27, 5-25.

Hoorn, C. 1994. Fluvial paleoenvironments in the intracratonic Amazonas Basin (Early Miocene-early Middle Miocene, Colombia). Palaeogeography, Palaeoclimatology, Palaeoecology 109, 1-54.

Hoorn, C., Kukla, T., Bogotá-Angel, G., van Soelen, E., González-Arango, C., Wesselingh, F.P., et al. 2022. Cyclic sediment deposition by orbital forcing in the Miocene wetland of western Amazonia? New insights from a multidisciplinary approach. Global and Planetary Change 210, 103717.

Jaramillo, C., Dilcher, D.L. 2001. Middle Paleogene palynology of Central Colombia, South America: a study of pollen and spores from tropical latitudes. Palaeontographica B 258, 87-213.

Lamy, A. 1986. Plio-Pleistocene palynology and visual kerogen studies, Trinidad, W.I., with emphasis on the Columbus Basin. Geological Society of Trinidad and Tobago, Geological Conference Transactions, pp. 114-127.

Langenheim, J.H., Hackner, B.L., Bartlett, A.S. 1967. Mangrove pollen at the depositional site of Oligo-Miocene amber from Chiapas, Mexico. Botanical Museum Leaflets Harvard University 21, 289-324.

Lorente, M.A. 1986. Palynology and palynofacies of the Upper Tertiary in Venezuela. Dissertationes Botanicae 99, 1-222.

Ochoa, D., Hoorn, C., Jaramillo, C., Bayona, G., Parra, M., De la Parra, F. 2012. The final phase of tropical lowland conditions in the axial zone of the Eastern Cordillera of Colombia: evidence from three palynoloogical records. Journal of South American earth Sciences 39, 157-169.

Parra, F.J., Navarrete, R.E., di Pasquo, M.M., Roddaz, M., Calderón, Y., Baby, P. 2020. Neogene palynostratigraphic zonation of the Maranon Basin, Western Amazonia, Peru. Palynology 44, 675-695.

Pocknall, D.T., Erlich, R.N. 2020. Palynostratigraphy and lithostratigraphy of Upper Cretaceous and Paleogene outcrop sections, Mérida Andes (Maracaibo Basin), Western Venezuela. Journal of South American Earth Sciences 104, 102830.

Pocknall, D.T., Wood, L.J., Geen, A.F., Harry, B.E., Hedlund, R.W. 2001. Integrated paleontological studies of Pliocene to Pleistocene deposits of the Orinoco Delta, Eastern Venezuela and Trinidad. In: Goodman, D.K., Clarke, R.T. (eds.), Proceedings of the IX International Palynological Congress, Houston, Texas. American Association of Stratigraphic Palynologists Foundation, pp. 319-326.

Regali, M., Uesugui, N., Santos, A. 1974. Palinologia dos sedimentos meso-cenozóicos do Brasil (I and II). Boletim Técnico PETROBRAS 17, 177-191, 263-301.

Regali, M.S.P., Uesugui, N., Lima, E.C. 1985. Palynostratigraphy and paleoenvironment of the Barreirinhas do Maranhao, Brazil. 7th Congreso Brasileiro de Paleontologia 27, 461-470.

Rodríguez-Forero, G., Oboh-Ikuenobe, F.E., Jaramillo-Munoz, C., Rueda-Serrano, M., Cadena-Rueda, E. 2012. Palynology of the Eocene esmeraldas Formation, Middle Magdalena Valley Basin, Colombia. Palynology 36, 96-111.

Rull, V. 1992. Paleoecología y análisis secuencial de una sección deltaica terciaria de la cuenca de Maracaibo. Boletín de la Sociedad Venezolana de Geólogos 46, 16-26.

Rull, V. 1997a. Oligo-Miocene palynology of the Rio Chama sequence (western Venezuela), with comments on fossil algae as paleoenvironemntal indicators. Palynology 21, 213-229.

Rull, V. 1997b. Sequence analysis of western Venezuelan Cretaceous to Eocene sediments using palynology: chronopaleoenvironmental and paleovegetational approaches. Palynology 21, 79-90.

Rull, V. 1998. Middle Eocene mangroves and vegetation changes in the Maracaibo Basin, Venezuela. Palaios 13, 287-296.

Rull, V. 1999. Palaeofloristic and palaeovegetational changes across the Paleocene/Eocene boundary in northern South America. Review of Palaeobotany and Palynology 107, 83-95.

Rull, V. 2000. Ecostratigraphic study of Paleocene and Early Eocene palynological cyclicity in northern South America. Palaios 15, 14-24.

Rull, V. 2001. A quantitative palynological record from the early Miocene of western Venezuela, with emphasis on mangroves. Palynology 25, 109–126.

Rull, V. 2002. High-impact palynology in petroleum geology: applications from Venezuela (northern South America). American Association of Petroleum Geologists Bulletin 86, 279-300.

Rull, V. 2003. Contribution of quantitative ecological methods to the interpretation of stratigraphically homogneous pre-Quaternary sediments: a palynological example from the Oligocene of Venezuela. Palynology 27, 75-98.

Rull, V., Poumot, C. 1997. Eocene to Miocene palynocycles from western Venezuela and correlations with global eustatic cycles. Memorias del VII Congreso Geológico Venezolano II, pp. 343-349.

Van der Hammen, T., Wijmstra, T.A. 1964. A palynological study on the tertiary and Upper Cretaceous of British Guiana. Ledise geologische Mededelingen 30, 183-241.

Wijmstra, T.A. 1968. The identity of *Psilatricolporites* and *Pelliciera*. Acta Botanica Neerlandica 17, 114-116.

Wijmstra, T.A. 1969. Palynology of the Alliance Well. Geologie en Mijnbouw 48, 125-133.
